# Optimal gender-specific age for cost-effective vaccination with adjuvanted herpes zoster subunit vaccine in Chinese adults

**DOI:** 10.1371/journal.pone.0210005

**Published:** 2019-01-04

**Authors:** Joyce H. S. You, Wai-kit Ming, Owen Tak-yin Tsang, Paul Kay-sheung Chan

**Affiliations:** 1 School of Pharmacy, Faculty of Medicine, The Chinese University of Hong Kong, Hong Kong SAR, China; 2 Harvard Medical School, Harvard University, Boston, Massachusetts, United States of America; 3 Department of Medicine & Geriatrics, The Princess Margaret Hospital, Hong Kong SAR, China; 4 Department of Microbiology, Faculty of Medicine, The Chinese University of Hong Kong, Hong Kong SAR, China; TNO, NETHERLANDS

## Abstract

**Background:**

Adjuvanted herpes zoster (HZ) subunit (HZ/su) vaccine is recommended for healthy adults aged ≥50 years, yet vaccine efficacy is expected to wane over time. Age-sex specific cost-effectiveness analyses of HZ/su vaccine are warranted to inform decision-making on vaccine policy. We aimed to determine the optimal gender-specific age for cost-effective HZ/su vaccination in Hong Kong.

**Methods:**

A Markov model was used to compare outcomes with and without HZ/su in healthy males and females at age 50–80 years. Model outcome measures were total cost, HZ cases, and HZ-associated quality-adjusted life-years (QALYs) loss. Incremental cost per QALY saved (ICER) by HZ/su was estimated for each age-sex group. Sensitivity analyses were performed to examine robustness of model results.

**Results:**

HZ/su reduced incidence of HZ in both males and females aged 50–80 years and the numbers needed to vaccinate to avoid one HZ case were lowest at age 60 years for males (6.05) and females (5.50). The highest QALY-saved occurred in females (0.00396 QALYs) and males (0.00379 QALYs) who were vaccinated at 60 years old. The ICERs were lowest at age 60–70 years for both genders. Using 1× gross domestic product per capita of Hong Kong (USD46,153) as willingness-to-pay threshold, HZ/su vaccine was accepted to be cost-effective for all female and male age groups at vaccine cost = USD160, for female aged 50–79 years and male aged 54–74 years at vaccine cost = USD200, and for female aged 59–71 years at vaccine cost = USD240.

**Conclusions:**

HZ/su vaccine is more likely to be cost-effective for males and females aged between 60–70 years than the extreme age groups (less than 60 years and older than 70 years) in Hong Kong. The age range for cost-effective acceptance of HZ/su vaccine appears to be broader in females than males given the same vaccine cost and willingness-to-pay threshold.

## Background

Herpes zoster (HZ), also known as shingles, is the reactivation of varicella zoster virus (VZV) infection. Primary VZV infection is commonly acquired in early stage of life and presented as chickenpox. The prominent symptoms of HZ are rash, pain and formation of vesicles on the affected dermatome. The most common complication of HZ is postherpetic neuralgia (PHN), occurring in 25% of HZ cases after resolution of pain and vesicles. The incidence of HZ in Taiwan Chinese rises sharply after the age of 50 years [1]. Both age and female gender are well-recognized risk factors for HZ [2]. The risk and duration of PHN increase with age [3,4]. Burden of disease study in Taiwan reported that the lifetime risk of developing HZ was over 30% in the Chinese population, and HZ-associated hospitalization rate also increased with age [1].

U.S. Food and Drug Administration (FDA) approved a live-attenuated zoster vaccine (ZVL) for 60 years and older individuals in 2006 and changed labelling to include those 50 years and older in 2011. ZVL reduced HZ cases in randomized clinical trials, yet further analysis showed that the vaccine efficacy (VE) in elderly (70 years or older) was significantly lower than the VE in the older adults (50–59 years) [5,6]. Adjuvanted HZ subunit vaccine (HZ/su), a 2-dose vaccine, was approved in 2017 by the U.S. FDA. The Advisory Committee on Immunization Practices (ACIP) of the Centers for Disease Control and Prevention (CDC) recommended HZ/su as the preferred vaccine against zoster for healthy adults aged 50 years and older as well as for adults who previously received ZVL [7]. Clinical findings showed the VE of HZ/su for adults aged 50 years and older to be over 95% [8]. Age-stratified analysis found the age-specific VEs to be higher than 90% across age groups from 50 to 80 years [8,9].

We previously developed a lifelong Markov model on HZ for Chinese subjects aged 50 years old, and found deferring HZ/su by 10 years was likely to be the preferred cost-effective strategy for a 50-year-old adult in Hong Kong [10]. The adult vaccination program subsidized by the Hong Kong government currently does not include vaccination against zoster. To further inform decision-making on vaccine policy, gender- and age-specific cost-effectiveness analyses of HZ/su vaccine are still warranted. This study therefore aimed to determine the optimal gender-specific age for the cost-effective vaccination with HZ/su from the societal perspective of Hong Kong.

## Methods

### Model design

**Fig 1:**
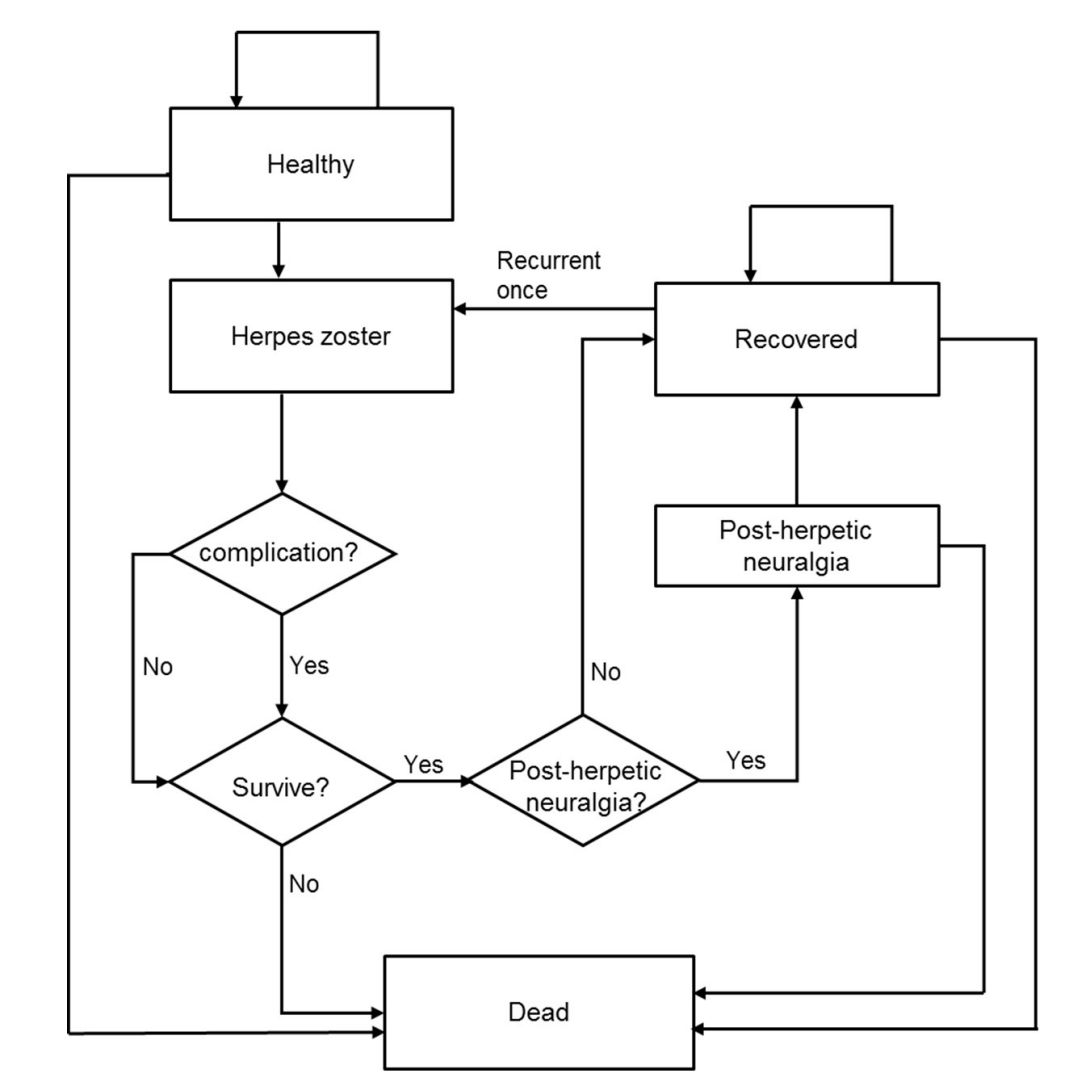
Markov diagram of herpes zoster.

A Markov decision analytic model on HZ, previously designed by our team [10], was adopted and further expanded to examine the life-long outcomes of each yearly age group from 50 years to 80 years for males and females separately (31 cohorts aged 50–80 years for each gender; total 62 cohorts). Costs and clinical outcomes, with HZ/su versus no vaccination, were compared for healthy males and females at age 50–80 years (**Fig 1**). All hypothetical subjects entered the model at the status of “healthy”. The subjects proceeded through health statuses according to probability inputs of the model in each monthly cycle. The health statuses included: Healthy, HZ, PHN, HZ recovered, recurrent HZ and death. PHN was defined as HZ-associated pain that was rated as ≥3 (on a scale from 0–10) persisting more than 90 days after the onset of HZ rash [5]. The outcomes of the subjects were simulated from the entry of model until the age reached 100 years or death, whichever occurred first. Model outcome measures were total cost, HZ cases, and HZ-associated quality-adjusted life-years (QALYs) loss.

A subject might survive or die of all causes in each cycle. With or without vaccination, a subject who survived in each cycle might develop HZ, and the HZ patient might receive outpatient care or become hospitalized. HZ patients who were hospitalized might develop HZ-related complications (central nervous system infection, ophthalmicus, oticus, skin and soft tissue infection, HZ dissemination). Patients who survived HZ might further develop PHN with various duration of pain. After the HZ episode (and PHN, if present) had resolved, the individual was assumed to be at risk for one episode of recurrent HZ.

### Model inputs

The retrieval of model inputs was described in our prior study [10]. In brief, clinical inputs were retrieved from published reports written in English on the incidence of HZ, PHN, HZ-related complication and mortality. Epidemiology or disease burden studies of HZ in Chinese population were preferred sources for baseline risk of HZ and PHN. Randomized controlled trial was the preferred type of study on HZ/su. Recurrent of HZ was not included in our prior HZ model, and we had incorporated HZ recurrence and subsequent HZ-related events in the present model. The recurrence rate of HZ in long term was reported in a retrospective observational study of 1,669 patients with history of HZ over an average follow-up period of 7.3 years. The findings suggested that incidences of HZ recurrence and first-episode HZ were similar [11]. The risk of one-time HZ recurrence therefore adopted the age-sex specific annual incidence of HZ. The full list of model inputs is available in supporting information (S1 Table).

The QALY loss for HZ/su-associated adverse event, HZ, PHN, or HZ-related death was calculated using the time spent in each health status, the corresponding disutility, and age-sex specific utility value for usual health. The QALY loss of HZ-associated death was projected using the age at death, life expectancy from lifetable, and age-sex specific utility value. The QALY loss for all events were discounted annually by 3%.

Direct and indirect costs were included in the cost analysis, conducted from the societal perspective of Hong Kong. Direct medical costs were costs of treatment for HZ, HZ complications and PHN in Hong Kong. The cost items included medications, laboratory tests, hospitalization, and outpatient clinic visits, and the calculation of costs was described previously [10]. HZ/su is not yet available in Hong Kong. Three levels of vaccine cost for the 2-dose HZ/su were examined by base-case analysis in reference to the vaccine cost of ZVL in Hong Kong: 1-, 1.25- and 1.5-fold of ZVL cost (USD160, USD200 and USD240, respectively; USD1 = HKD7.8). The treatment cost for injection site reaction included costs of over-the-counter analgesic and antipyretics. Indirect costs of HZ and related events were estimated by the friction cost approach using age- and sex-specific labor force participation rates, unemployment rates and median monthly incomes. Patient-time used to seek inpatient or outpatient care was applied as friction period of the corresponding event. All costs were discounted annually by 3%.

### Cost-effectiveness analysis and sensitivity analyses

Incidence of HZ, QALY loss and costs were calculated with and without vaccination in base-case analysis for males and females over age 50–80 years at three levels of vaccine cost. The incremental cost-effectiveness ratio (ICER) of HZ/su was calculated by using the equation: (Total cost_vaccine_−Total cost_no vaccine_)/(QALY loss_no vaccine_−QALY_loss vaccine_). The World Health Organization (WHO) recommended that ICER less than 1× gross domestic product (GDP) per capita to be highly cost-effective [12]. The GDP per capita of Hong Kong was USD46,153 in 2017 [13], and it was adopted as the threshold of willingness-to-pay (WTP). The ICER less than USD46,153 (1× GDP per capita) were accepted as cost-effective in the present analysis.

Sensitivity analyses were performed by one-way sensitivity analysis and probabilistic sensitivity analysis (using Monte Carlo simulation). The probability of HZ/su vaccination to be accepted as cost-effective was determined over a range of WTP from 0–100,000 USD/QALY in the acceptability curves. Cost-effectiveness and sensitivity analyses were conducted by TreeAge Pro 2009 (TreeAge Software, Inc., Williamstown, MA, USA) and Microsoft Excel 2010 (Microsoft Corporation, Redmond, WA, USA).

## Results

### Model validation

The predictive validity of model was examined by comparing the life-long HZ incidence of 50-year-old male and female in no vaccination group with lifetime risk of developing HZ in Chinese population. The non-gender specific lifetime risk of one occurrence of HZ in Taiwan Chinese was reported to be 32.2% [1]. Using zero as input for probability of HZ recurrence and 52.6% as proportion of female gender in 50-year-old Hong Kong population [14], the expected life-long incidence of 50-year-old populated was 30.0%. The relative difference between expected lifetime HZ incidence and reported incidence was less than 7%.

### Base-case analysis

**Fig 2:**
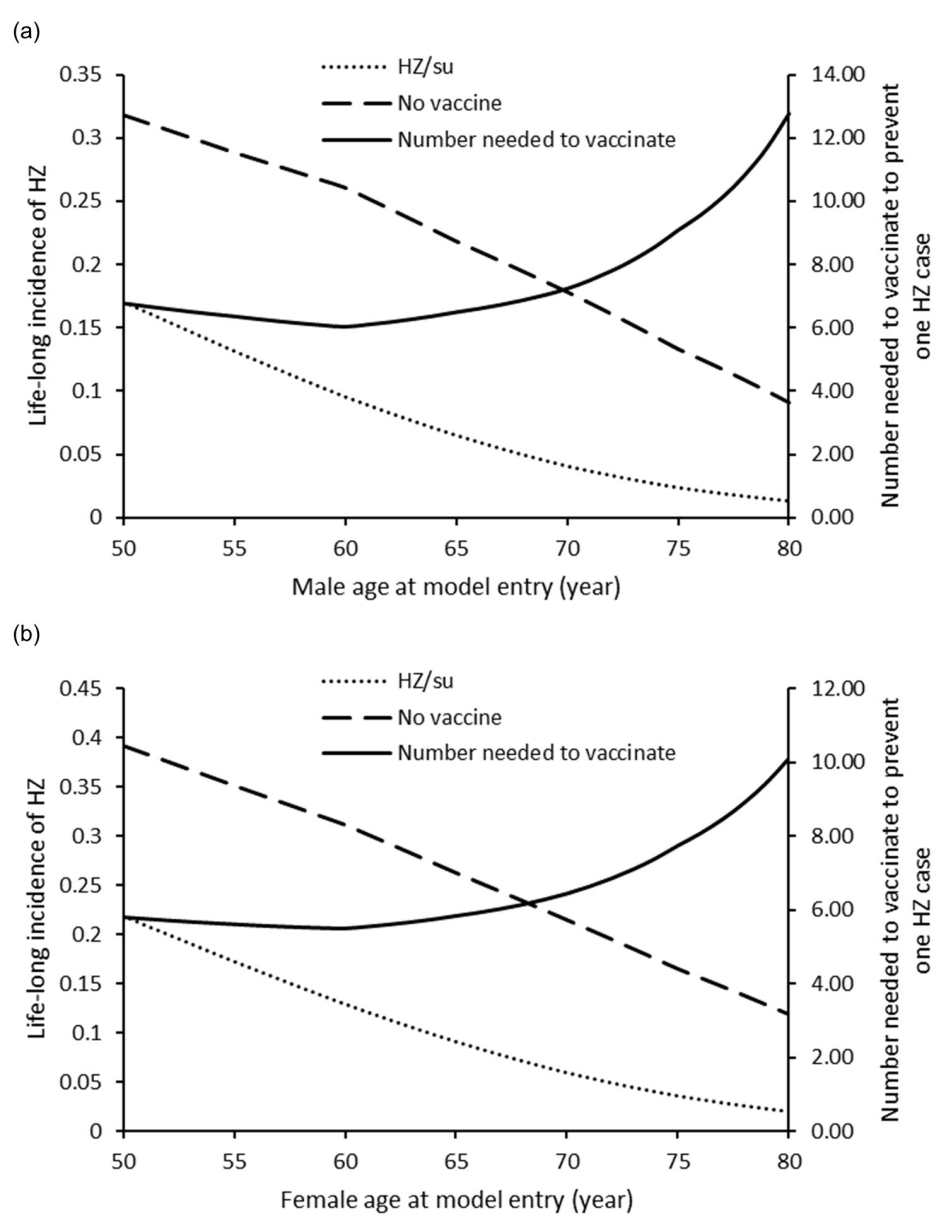
**Change of HZ incidence with HZ/su and no vaccine and number needed to vaccinate against vaccination age in Chinese (a) males and (b) females**.

HZ/su reduced incidence of HZ in both genders at age 50–80 years. The numbers needed to vaccinate to avoid one HZ case were lowest at age 60 years in males (6.05) and females (5.50) in the base-case analysis (**Fig 2A and 2B**). The cases of HZ avoided by HZ/su vaccine were higher in females than males for all age groups.

**Fig 3:**
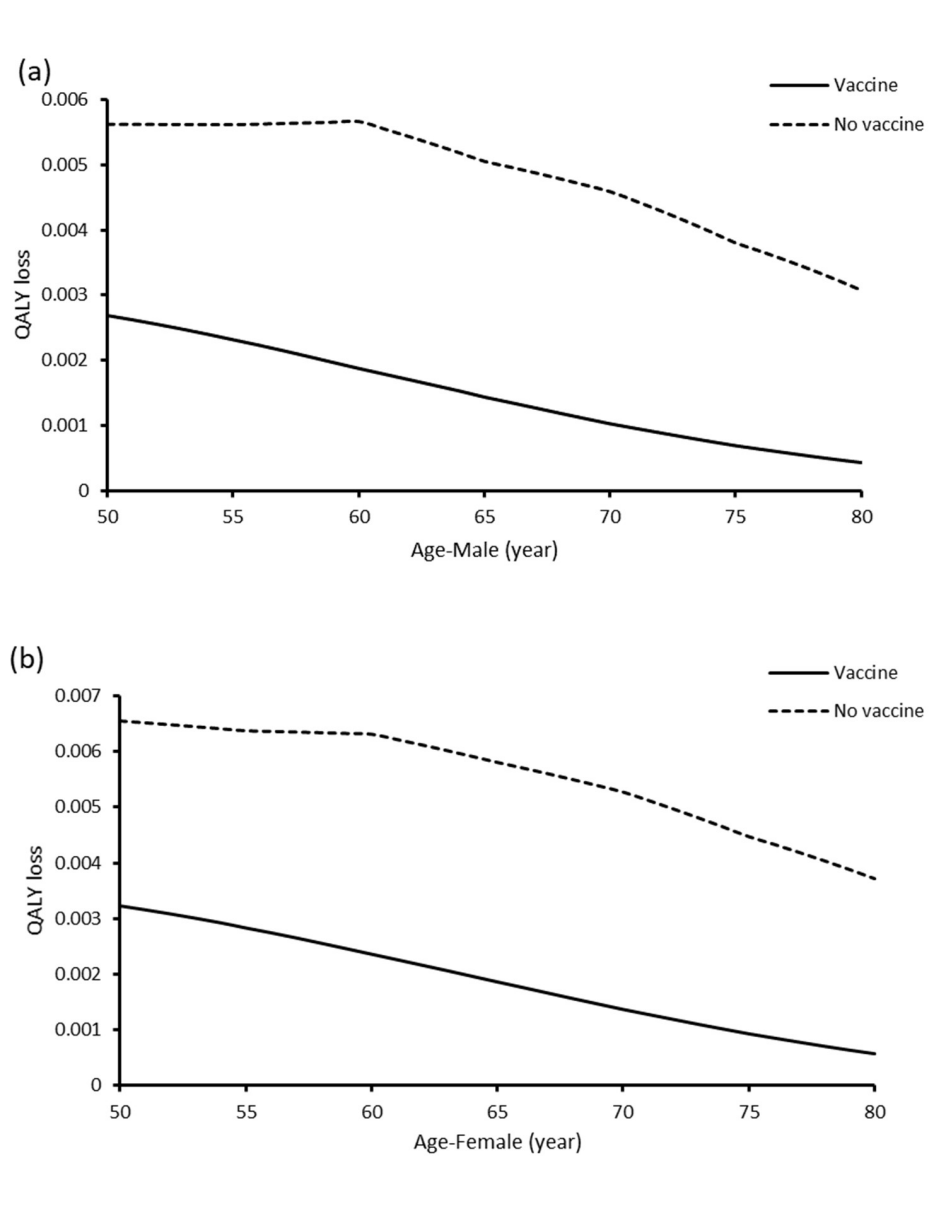
**Change of QALY loss with HZ/su and no vaccine against vaccination age in (a) males and (b) females**.

**Fig 4:**
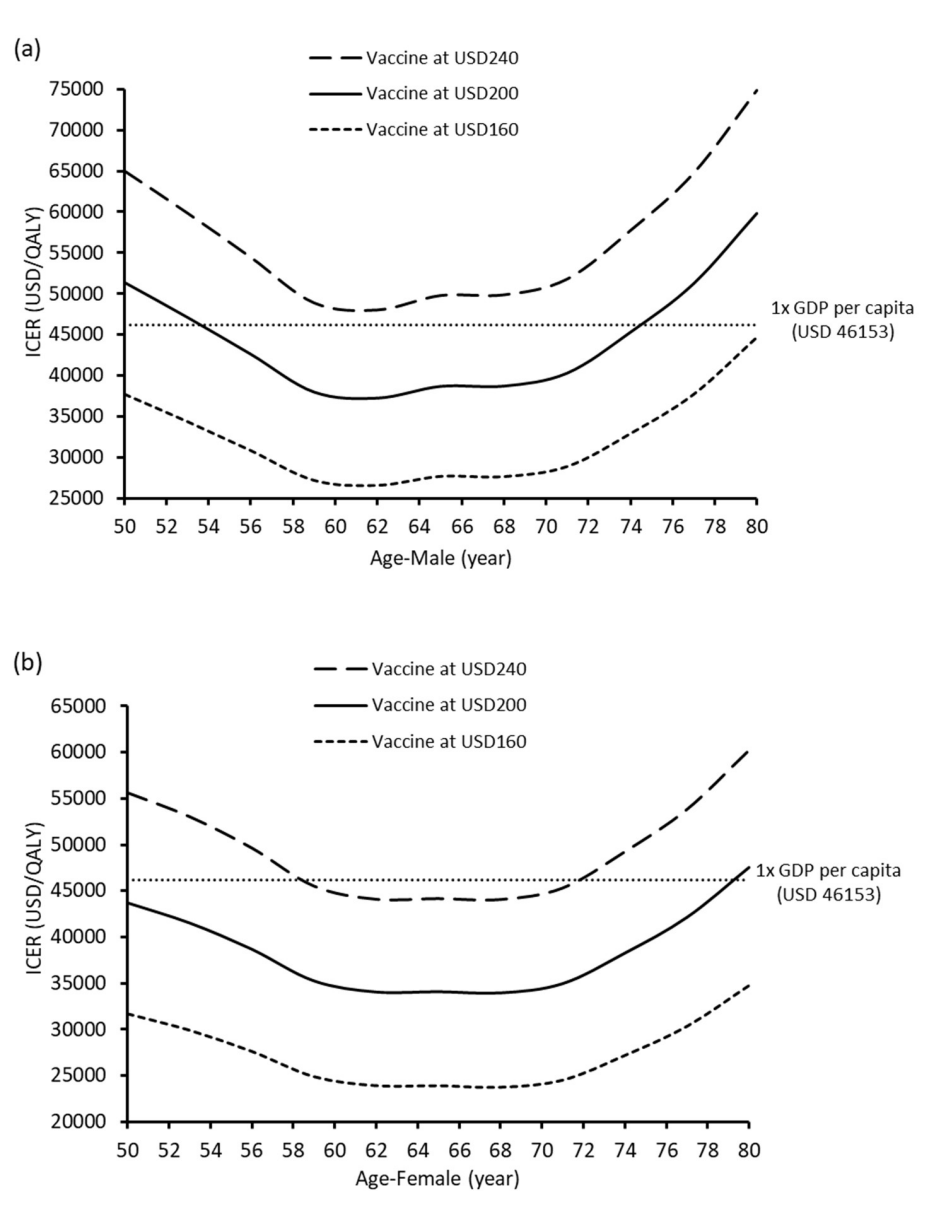
**Change of incremental cost-effectiveness ratio (ICER) of HZ/su against vaccination age at three vaccine costs in (a) males and (b) females**.

The QALY loss in the HZ/su and no vaccine arms at age 50–80 years for males and females were shown in **Fig 3A and 3B.** The QALYs saved by HZ/su was higher in females than males of all age groups. The highest QALY-saved occurred in females (0.00396 QALYs) and males (0.00379 QALYs) who were vaccinated at 60 years old. The age-sex specific ICERs of HZ/su at each vaccine cost were lowest at age 60–70 years (**Fig 4A and 4B).** At vaccine cost of USD160, HZ/su was accepted as cost-effective for males and females aged 50–80 years. At vaccine cost of USD200, HZ/su was cost-effective in females aged 50–79 years and males aged 54–74 years. At vaccine cost of USD240, HZ/su was cost-effective only in females aged 59–71 years.

### Sensitivity analysis

Using vaccine cost USD200 and 60-year-old as base-case inputs, one-way sensitivity analysis on all model inputs for each gender group was performed and two influential factors were identified. Age was an influential factor in both gender groups and vaccine cost was an influential in the male group. In the male group, the ICER of HZ/su remained below the WTP threshold if the subject received HZ/su vaccine at age 54–74 years, or if the vaccine cost was less than USD236. In the female group, the ICER of HZ/su was less than the WTP threshold if the subject received HZ/su vaccine at age ≤79 years.

The probabilistic sensitivity analysis was performed by 10,000 Monte Carlo simulations at vaccine cost of USD200 for four age groups (50 years, 60 years, 70 years and 80 years) of the two genders. The QALYs saved in females who were vaccinated at age 50 years, 60 years, 70 years and 80 years were 0.00342 (95%CI 0.00341–0.00343), 0.00409 (95%CI 0.00408–0.00410), 0.00408 (95%CI 0.00408–0.00409) and 0.00352 (95%CI 0.00351–0.00353), respectively. The incremental cost was highest in females aged 80 years (USD138.4; 95%CI 138.1–138.6), followed by 50 years (USD127.0; 95%CI 126.6–127.4), 70 years (USD119.0; 95%CI 118.7–119.4) and 60 years (USD117.8; 95%CI 117.4–118.2). Using 1× GDP per capita as WTP threshold, percentages of simulations with ICER less than WTP were 85.5% in 50 years, 99.7% in 60 years, 99.7% in 70 years and 77.0% in 80 years in the female group.

In the male group, QALYs saved in subjects who were vaccinated at age 50 years, 60 years, 70 years and 80 years were 0.00300 (95%CI 0.00299–0.00301), 0.00389 (95%CI 0.00388–0.00390), 0.00368 (95%CI 0.00367–0.00369) and 0.00294 (95%CI 0.00293–0.00294), correspondingly. The incremental cost was highest in males aged 80 years (USD150.4; 95%CI 150.2–150.6), followed by 50 years (USD132.1; 95%CI 131.8–132.4), 70 years (USD127.4; 95%CI 127.1–127.7) and 60 years (USD122.0; 95%CI 121.7–122.4). The percentages of simulations with ICER less than 1× GDP per capita were 57.9% in 50 years, 98.6% in 60 years, 95.6% in 70 years and 26.5% in 80 years.

**Fig 5:**
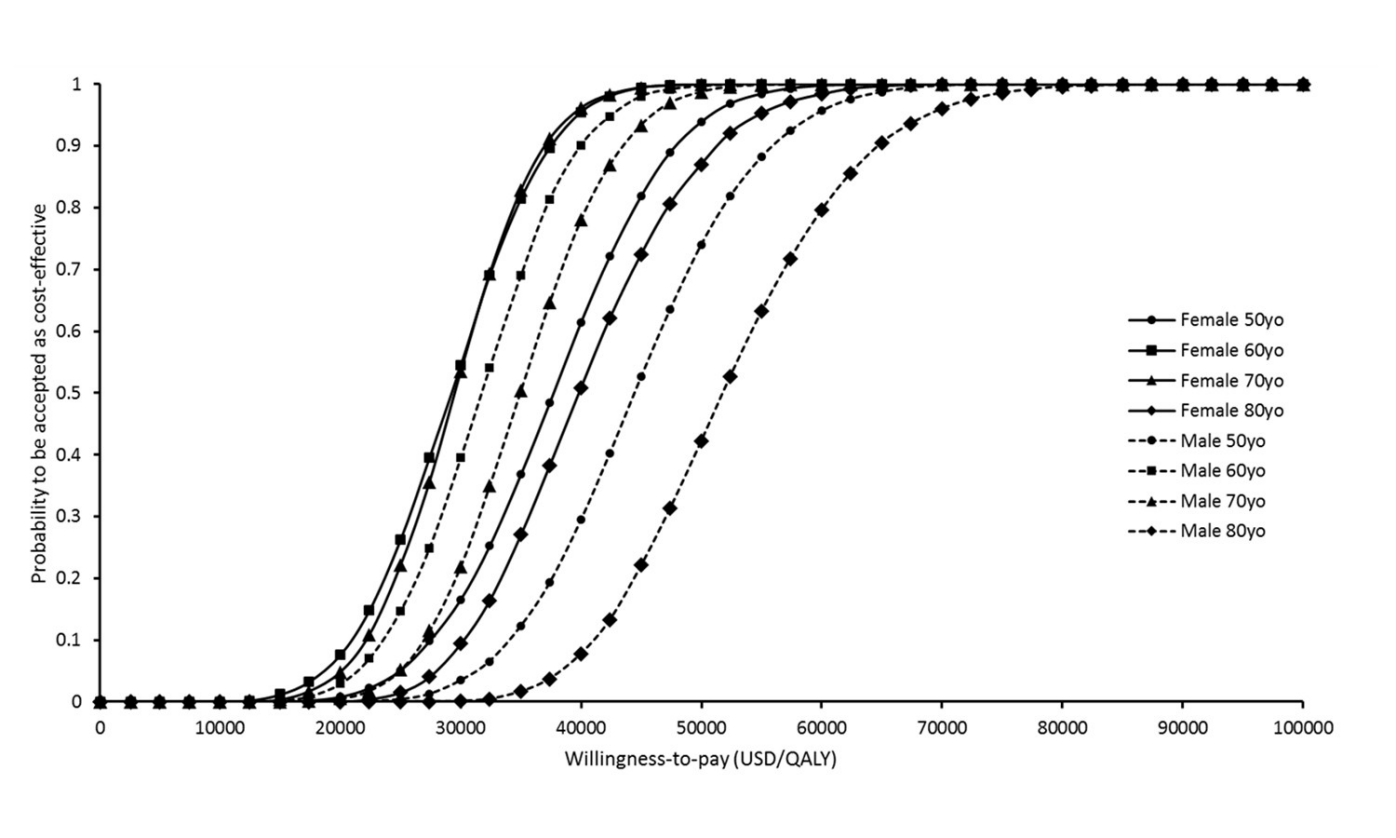
Variation in probabilities of HZ/su to be cost-effective against willingness-to-pay per QALY saved in males and females aged 50 years, 60 years, 70 years and 80 years.

The probabilities of HZ/su vaccination to be accepted as cost-effective in each age-gender group were showed in the acceptability curves over a wide range of WTP (0-USD100,000 USD/QALY) (**Fig 5**).

## Discussion

The HZ/su vaccine saved QALYs for all age-sex groups at higher cost, and the vaccine gained the highest QALYs in age 60-year for both gender groups. Comparing between male and female groups, higher QALY-saving and lower additional cost were consistently found in female groups aged 50–80 years. The base-case analysis found the acceptance of HZ/su vaccine to be cost-effective subject to the vaccine cost, gender and age. The sensitivity analysis results further supported the robustness of base-case findings. Of 10,000 Monte Carlo simulations for different age-sex groups, the probability of the HZ/su vaccine ICER to be <1× GDP per capita exceeded 90% in females and males aged 60 years and 70 years. The acceptability curves over a wide-range of WTP showed that the 60-year and 70-year groups are more likely to be accepted as cost-effective than the 50-year or 80-year groups at WTP below 80,000 USD/QALY.

A cost-effectiveness analysis of HZ/su at age 60 years, 70 years and 80 years (versus no vaccine) in the United States reported that the ICERs of older groups (70 years and 80 years) were lower than the ICER of younger age group (60 years), and the HZ/su vaccine was more likely to be cost-effective in older population [15]. Similarly, the present study found the ICERs of HZ/su vaccine in older age (60 years and 70 years) groups of both genders were lower than the 50-year groups. Different to the US findings, our results showed the ICER of HZ/su vaccine in 80-years group to be the highest. In the US study, the model inputs of HZ incidence were highest for the age groups ≥80 years. The model inputs of HZ incidence in Chinese population were highest at age 60–69 years and 70–79 years. In addition to the difference in age-specific HZ incidence, the US study allowed the hypothetical subject to reach a maximum age of 120 years, and the present model ended when the subject reached 100 years. With higher HZ incidence for advanced age and longer model time horizon, the US cost-effectiveness model therefore generated higher number of HZ cases avoided, resulting in higher QALYs gained and lower ICER at advanced age.

A model-based analytical study in the US recently reported that the most cost-effective age groups to receive HZ/su were 66–77 years for females and 66–74 years for males at vaccine cost (including administration) of USD205 [16]. Our results were similar to the US findings that the age groups 60–70 years had the lowest ICERs in both genders, and females had a wider range of optimal age to be vaccinated than males at the same vaccine cost in Hong Kong.

Hong Kong is a city of China and the Chinese population in Hong Kong have life-expectancy beyond 80 years for men and women. Over 50% of the seven million Hong Kong population were females and the age of 40% of the entire population were 50 years or above [14]. With increasing size of aging population and higher proportion of female-gender, the disease burden of HZ in Hong Kong is expected to rise. Exposure to varicella boosts immunity to HZ, and vaccination against varicella in children might increase the HZ incidence in adults due to the reduced exogenous boosting effect from children shedding VZV in the community [17]. Universal varicella vaccination was recommended in the US for children in May 1995, and both the charges and population-adjusted rates of HZ-related hospital discharges had increased significantly by 2004 [18]. The 2014 implementation of universal varicella vaccination for children in Hong Kong might possibly increase the HZ incidence. On the other hand, the vaccination program is anticipated to be costly if HZ/su is offered across population aged 50 years and above. The current dilemma for public healthcare providers and the government to decide is how to use the HZ/su vaccine cost-effectively, balancing the at-risk gender and age groups, persistence of vaccine efficacy, cost of vaccination and cost savings from prevented cases. Our findings provided important information to clinicians and the government on the decision-making process of zoster vaccine policy to maximize cost-effectiveness of the vaccination program in Hong Kong.

There are a number of limitations in the present analysis. The model simplified real-life events of HZ. Long-term disabilities such as deafness from oticus and blindness from ophthalmicus were not included in the model. Also, there is a growing body of emerging data suggesting an increased risk of stroke and myocardial infarction/transient ischemic attack associated with HZ [19–23], and the cardiovascular and cerebrovascular events following HZ were not included in the model. The lifelong QALYs saved by HZ/su might therefore be underestimated. The literature search for model clinical inputs was limited to publications written in English. Reports written in other language (such as Chinese language) may provide relevant inputs to the model. The waning of vaccine efficacy was extracted from manufacturer’s information reported in ACIP meeting, and impact of booster dose was not assessed in the model. Rare but severe complications of HZ was not included in the model. Also, the loss of productivity for caregivers of HZ patients was not included and might therefore underestimated the indirect cost saved by vaccination.

## Conclusions

HZ/su vaccine is more likely to be accepted as cost-effective for both genders aged between 60–70 years than the extreme age groups (less than 60 years and older than 70 years) in Hong Kong. The probabilities of vaccine to be cost-effective are higher in females than males for the same age group. The age range for cost-effective acceptance of HZ/su vaccine was broader in females than males given the same vaccine cost and cost-effectiveness threshold.

## Supporting information

S1 Table Model input. (DOCX)
