# Supplementary material for: Optimal gender-specific age for cost-effective vaccination with adjuvanted herpes zoster subunit vaccine in Chinese adults
Source: PLoS One. 2019 Jan 4;14(1):e0210005. doi: 10.1371/journal.pone.0210005 (PMC6319773; doi:10.1371/journal.pone.0210005)
Supplement: S1 Table — (DOCX) [file pone.0210005.s001.docx]

**S1 Table. Model inputs.**

|  | **Base-case value (Range for sensitivity analysis)** | | | | | | | | | | | | | | **Distribution** | **Reference** |
| --- | --- | --- | --- | --- | --- | --- | --- | --- | --- | --- | --- | --- | --- | --- | --- | --- |
| **Clinical parameters** | | | | | | | | | | | | | | | | |
| ***Gender specific parameters*** | | | | | | | | | | | | | | | | |
| **Male** | Age (years) | | | | | | | | | | | | | |  |  |
|  | 50-54 | 55-59 | | 60-64 | 65-69 | | 70-74 | 75-79 | | | | 80-84 | | ≥85 |  |  |
| All-cause mortality rates per 1000 people aged | 3.2  (3.1-3.7) | 5.1  (5.1-5.6) | | 8.1  (8.1-9.9) | 12.5  (12.2-15.4) | | 21.1  (21.1-26.8) | 34.8  (34.8-44.1) | | | | 61.5  (61.5-76.0) | | 127.5  (127.5-129.6) | Beta | 1 |
| Annual HZ incidence per 1000 people aged | 7.2  (5.8-8.6) | | | 10.7  (8.6-12.8) | | | 12.5  (10.0-15.0) | | | | | 10.0  (8.0-12.0) | | | Beta | 2 |
| Proportion of zoster cases with PHN in patients aged | 7.07%  (5.66-8.48%) | | | 15.42%  (12.34-18.50%) | | | | | | | | 31.53%  (25.22-37.84%) | | | Beta | 3 |
| **Female** | Age (years) | | | | | | | | | | | | | |  |  |
|  | 50-54 | 55-59 | | 60-64 | 65-69 | | 70-74 | 75-79 | | | | 80-84 | | ≥85 |  |  |
| All-cause mortality rates per 1000 people aged | 1.8  (1.6-2.0) | 2.7  (2.6-2.8) | | 3.9  (3.9-4.4) | 6.1  (5.8-6.9) | | 9.7  (9.7-13.1) | 18.6  (18.4-23.8) | | | | 34.9  (34.9-43.3) | | 92.2  (90.4-98.2) | Beta | 1 |
| Annual HZ incidence per 1000 people aged | 9.3  (7.4-11.2) | | | 11.3  (9.0-13.6) | | | 12.0  (9.6-14.4) | | | | | 10.5  (8.4-12.6) | | | Beta | 2 |
| Proportion of zoster cases with PHN in patients aged | 6.94%  (5.55-8.33%) | | | 13.53%  (10.82-16.24%) | | | | | | | | 21.9%  (17.52-26.28%) | | | Beta | 3 |
| ***Non-gender specific parameters*** | | | | | | | | | | | | | | |  |  |
|  | Age (years) | | | | | | | | | | | | | |  |  |
|  | 50-54 | 55-59 | | 60-64 | 65-69 | | 70-74 | 75-79 | | | | 80-84 | | ≥85 |  |  |
| Hospitalization rate among HZ patients aged | 2.67%  (2.14-3.20%) | | | 4.14%  (3.31-4.97%) | | | 7.17%  (5.74-8.60%) | | | | | 10.29%  (8.23-12.3%) | | | Beta | 2 |
| HZ-associated mortality rate among hospitalized patients aged | 0.056%  (0-0.133%) | | | 0.111%  (0.077-0.130%) | | | 0.293%  (0.209-0.378%) | | | | | 1.151%  (0.546-3.578%) | | | Beta | 4 |
| HZ complication rate among hospitalized patients | | | | | | | | | | | | | | | Beta | 5 |
| Ophthalmicus | | | | 6.7% (3.3-13.1%) | | | | | | | | | | |  |  |
| Skin and soft tissue infection | | | | 3.8% (1.5-9.4%) | | | | | | | | | | |  |  |
| Oticus | | | | 1.9% (0.5-6.7%) | | | | | | | | | | |  |  |
| HZ dissemination | | | | 1% (0.2-5.2%) | | | | | | | | | | |  |  |
| Central nervous system infection | | | | 1% (0.2-5.2%) | | | | | | | | | | |  |  |
| Monthly probability of PHN to persist:  P(*t*)=1−exp[λ(*t*−1)^γ^ − λ*t* ^γ^] | | | | λ | | | | | γ | | | | | | - | 6,7 |
| Age <60 | | | | 0.06203 | | | | | 0.51101 | | | | | |  |  |
| Age ≥60 | | | | 0.24885 | | | | | 0.46843 | | | | | |  |  |
| Vaccine efficacy | | | | 2 doses | | | | | 1 dose | | | | | |  | 8,9,10 |
| Efficacy function intercept | | | | 1.0765 | | | | | 0.8801 | | | | | |  |  |
| Annual waning rate | | | | 0.0319 (0.0255-0.0383) | | | | | 0.0507 (0.0406-0.0608) | | | | | | Triangular |  |
| Vaccine uptake | | | | 1^st^ dose | | | | | 2^nd^ dose | | | | | |  |  |
|  | | | | 100% (0-100%) | | | | | 100% (0-100%) | | | | | | Uniform |  |
| Duration of injection-site reaction (days) | | | | 2 (1-3) | | | | | | | | | | | Triangular | 8 |
| **Utility parameters** | | | | | | | | | | | | | | | | |
| ***Gender specific parameters*** | | | | Age (years) | | | | | | | | | | |  |  |
| Usual health utility at age | | | | 50-54 | | | 55-64 | | | | >65 | | | | - | 11 |
| Male | | | | 0.92 | | | 0.92 | | | | 0.84 | | | |  |  |
| Female | | | | 0.92 | | | 0.84 | | | | 0.84 | | | |  |  |
| ***Non-gender specific parameters*** | | | |  | | | | | | | | | | |  |  |
| Disutility | | | | | | |  | | | | | | | | Triangular |  |
| Injection site reaction | | | | | | | 0.01 (0.005-0.015) | | | | | | | |  | Assumption |
| Outpatient zoster care | | | | | | | 0.31 (0.25-0.37) | | | | | | | |  | 12 |
| Inpatient care without zoster complication | | | | | | | 0.42 (0.34-0.50) | | | | | | | |  | 12 |
| Inpatient care with zoster complication | | | | | | | 0.75 (0.60-0.90) | | | | | | | |  | 12 |
| PHN | | | | | | | 0.42 (0.31-0.75) | | | | | | | |  | 12 |
| **Cost parameters** | | | | | | | | | | | | | | | | |
| Direct cost inputs (USD) (USD1=HKD7.8) | | | | | | |  | | | | | | | |  |  |
| HZ/su vaccine (2 doses) | | | | | | | 160; 200; 240 | | | | | | | | - | Estimation |
| Injection site reaction | | | | | | | 1 (0.5-2) | | | | | | | | Uniform | Local prices |
| Cost per HZ case | | | | | | |  | | | | | | | | Gamma | 5 |
| Inpatient HZ care with no complication or PHN | | | | | | | 2887 (1930-4816) | | | | | | | |  |  |
| Ophthalmicus | | | | | | | 3068 (1938-4932) | | | | | | | |  |  |
| Skin and soft tissue infection | | | | | | | 1900 (1292-4386) | | | | | | | |  |  |
| Oticus | | | | | | | 6597 (2532-10662) | | | | | | | |  |  |
| HZ dissemination | | | | | | | 6299 (5039-7558) | | | | | | | |  |  |
| Central nervous system infection | | | | | | | 26269 (21015-31523) | | | | | | | |  |  |
| Outpatient care with no complication or PHN | | | | | | | 309 (144-813) | | | | | | | |  |  |
| Cost per case per month (with PHN) | | | | | | | 40 (32-115) | | | | | | | |  |  |
| Indirect costs | | | | | | | | | | | | | | |  |  |
| ***Gender specific parameters*** | | | | | | | | | | | | | | |  |  |
| **Male** | | | Age (years) | | | | | | | | | | | |  |  |
|  |  |  | 50-54 | | | 55-59 | | | | 60-64 | | | ≥65 | |  |  |
| Labor force participation rate at age | | | 90.8%  (76.6-100%) | | | 81.4%  (65.1-97.7%) | | | | 60.5%  (48.4-72.6%) | | | 15.6%  (12.5-18.7%) | | Beta | 1 |
| Unemployment rate at age | | | 3.2% (2.6-3.8%) | | | | | | | 2.3% (1.8-2.8%) | | | | | Beta | 1 |
| Monthly income of employed persons at age (USD) | | | 2436 (1949-2923) | | | | | | | 1667 (1334-2000) | | | | | Triangular | 1 |
| **Female** | | | Age (years) | | | | | | | | | | | |  |  |
|  |  |  | 50-54 | | | 55-59 | | | | 60-64 | | | ≥65 | |  |  |
| Labor force participation rate at age | | | 66.0% (52.8-79.2%) | | | 50.2% (40.2-60.2%) | | | | 29.2% (23.4-35.0%) | | | 4.8% (3.8-5.8%) | | Beta | 1 |
| Unemployment rate at age | | | 2.8% (2.2-3.4%) | | | | | | | 2.0% (1.6-2.4%) | | | | | Beta | 1 |
| Monthly income of employed persons at age (USD) | | | 1538 (1230-1846) | | | | | | | 1115 (892-1338) | | | | | Triangular | 1 |
| ***Non-gender specific parameters*** | | | | | | | | | | | | | | |  |  |
| Length of stay (days) | | | | | | | | | | | | | | | Triangular | 5 |
| Ophthalmicus | | | | | | | 5 (3-8) | | | | | | | |  |  |
| Skin and soft tissue infection | | | | | | | 3 (2-7) | | | | | | | |  |  |
| Oticus | | | | | | | 11 (4-17) | | | | | | | |  |  |
| HZ dissemination | | | | | | | 10 (8-12) | | | | | | | |  |  |
| Central nervous system infection | | | | | | | 43 (34-52) | | | | | | | |  |  |
| No complication/PHN | | | | | | | 5 (3-8) | | | | | | | |  |  |
| Number of outpatient visit for case with no complication/PHN | | | | | | | 1 (1-2) | | | | | | | |  |  |

**Reference:**

1. Census and Statistics Department, Hong Kong SAR. Women and men in Hong Kong key statistics 2017 Edition. Website: <https://www.censtatd.gov.hk/hkstat/sub/sp180.jsp?productCode=B1130303> Assessed on 3 January 2018
2. Lin YH, Huang LM, Chang IS, Tsai FY, Lu CY, Shao PL, et al. Disease burden and epidemiology of herpes zoster in pre-vaccine Taiwan. Vaccine 2010;28:1217-1220.
3. Jih JS, Chen YJ, Lin MW, Chen YC, Chen TJ, Huang YL, et al. Epidemiological features and costs of herpes zoster in Taiwan: A national study 2000 to 2006. Acta Derm Venereol 2009;89:612-616.
4. Ultsch B, Siedler A, Rieck T, Reinhold T, Krause G, Wichmann O. Herpes zoster in Germany: quantifying the burden of disease. BMC Infect Dis. 2011;11:173.
5. You JHS, Ming WK, Lee CF, Tsang OT, Chan PK. Potential cost-effectiveness of adjuvanted herpes zoster subunit vaccine for older adults in Hong Kong. Vaccine 2018; 25;36:4610-4620.
6. Helgason S, Petursson G, Gudmundsson S, Sigurdsson JA. Prevalence of postherpetic neuralgia after a first episode of herpes zoster: prospective study with long term follow up. BMJ. 2000;321:794-6.
7. A. Briggs, M. Sculpher, K. Claxton, Decision Modelling for Health Economic Evaluation, 1st ed., Oxford University Press, 2006.
8. Lal H, Cunningham AL, Godeaux O, Chlibek R, Diez-Domingo J, Hwang SJ, et al. Efficacy of an adjuvanted herpes zoster subunit vaccine in older adults. N Engl J Med. 2015;372:2087-96.
9. Cunningham AL, Lal H, Kovac M, Chlibek R, Hwang SJ, Díez-Domingo J, et al. Efficacy of the Herpes Zoster Subunit Vaccine in Adults 70 Years of Age or Older. N Engl J Med. 2016;375:1019-32.
10. Overview of two economic models that assess the cost-effectiveness of herpes zoster vaccinations. Advisory Committee on Immunization Practices June 21, 2017. Website: <https://www.cdc.gov/vaccines/acip/meetings/downloads/slides-2017-06/zoster-04-leidner.pdf> Assessed on 9 November 2017
11. Gold MR, Franks P, McCoy KI, Fryback DG. Toward consistency in cost-utility analyses: using national measures to create condition-specific values. Med Care 1998;36:778-92.
12. Oster G, Harding G, Dukes E, Edelsberg J, Cleary PD. Pain, medication use, and health-related quality of life in older persons with postherpetic neuralgia: results from a population-based survey. J Pain. 2005;6:356-63.
